# Genome-Wide Scan Informed by Age-Related Disease Identifies Loci for Exceptional Human Longevity

**DOI:** 10.1371/journal.pgen.1005728

**Published:** 2015-12-17

**Authors:** Kristen Fortney, Edgar Dobriban, Paolo Garagnani, Chiara Pirazzini, Daniela Monti, Daniela Mari, Gil Atzmon, Nir Barzilai, Claudio Franceschi, Art B. Owen, Stuart K. Kim

**Affiliations:** 1 Department of Developmental Biology, Stanford University, Stanford, California, United States of America; 2 Department of Genetics, Stanford University, Stanford, California, United States of America; 3 Department of Statistics, Stanford University, Stanford, California, United States of America; 4 Department of Experimental, Diagnostic and Specialty Medicine Experimental Pathology, University of Bologna, Bologna, Italy; 5 Center for Applied Biomedical Research, St. Orsola-Malpighi University Hospital, Bologna, Italy; 6 Interdepartmental Centre "L. Galvani" CIG, University of Bologna, Bologna, Italy; 7 Department of Clinical, Experimental and Biomedical Sciences, University of Florence, Florence, Italy; 8 Department of Medical Sciences, University of Milan, Milan, Italy; 9 Geriatric Unit, IRCCS Ca' Grande Foundation, Maggiore Policlinico Hospital, Milan, Italy; 10 Department of Genetics, Albert Einstein College of Medicine, Bronx, New York, United States of America; 11 IRCCS, Institute of Neurological Sciences of Bologna, Bologna, Italy; University of California - San Francisco, UNITED STATES

## Abstract

We developed a new statistical framework to find genetic variants associated with extreme longevity. The method, informed GWAS (iGWAS), takes advantage of knowledge from large studies of age-related disease in order to narrow the search for SNPs associated with longevity. To gain support for our approach, we first show there is an overlap between loci involved in disease and loci associated with extreme longevity. These results indicate that several disease variants may be depleted in centenarians versus the general population. Next, we used iGWAS to harness information from 14 meta-analyses of disease and trait GWAS to identify longevity loci in two studies of long-lived humans. In a standard GWAS analysis, only one locus in these studies is significant (*APOE/TOMM40*) when controlling the false discovery rate (FDR) at 10%. With iGWAS, we identify eight genetic loci to associate significantly with exceptional human longevity at FDR < 10%. We followed up the eight lead SNPs in independent cohorts, and found replication evidence of four loci and suggestive evidence for one more with exceptional longevity. The loci that replicated (FDR < 5%) included *APOE/TOMM40* (associated with Alzheimer’s disease), *CDKN2B/ANRIL* (implicated in the regulation of cellular senescence), *ABO* (tags the O blood group), and *SH2B3/ATXN2* (a signaling gene that extends lifespan in *Drosophila* and a gene involved in neurological disease). Our results implicate new loci in longevity and reveal a genetic overlap between longevity and age-related diseases and traits, including coronary artery disease and Alzheimer’s disease. iGWAS provides a new analytical strategy for uncovering SNPs that influence extreme longevity, and can be applied more broadly to boost power in other studies of complex phenotypes.

## Author Summary

Longevity is a complex phenotype, and few genetic variants that affect lifespan have been identified. However, aging and disease are closely related, and a great deal is known about the genetic basis of disease risk. Here, we show using genome-wide association studies (GWAS) of longevity and disease that there is an overlap between loci involved in longevity and loci involved in several diseases, such as Alzheimer’s disease and coronary artery disease. We then develop a new statistical framework to find genetic variants associated with extreme longevity. The method, informed GWAS (iGWAS), takes advantage of knowledge from 14 large studies of disease and disease-related traits in order to narrow the search for SNPs associated with longevity. Using iGWAS, we found eight SNPs that are significant in our discovery cohorts, and we were able to validate four of these in replication studies of long-lived subjects. Our results implicate new loci in longevity and reveal a genetic overlap between longevity and age-related diseases and traits. Beyond the study of human longevity, iGWAS can be applied to boost statistical power in any GWAS of a target phenotype by using larger GWAS of genetically-related conditions.

## Introduction

Aging and disease are closely related as aging is the largest risk factor for multiple chronic diseases. A great deal is known about the genetic basis of disease risk from genome-wide-association studies. However, little is known about the specific genetic differences that contribute to varying life expectancies between individuals. The heritability of survival to the late eighties is estimated to be ~25–30% from twin studies[1], and the genetic influence on survival increases with increasing age beyond the top 5^th^ percentile of survival[2–4].

To find specific loci for longevity, genetic association studies have been used to identify polymorphisms that are associated with exceptional longevity. The first studies tested candidate genes suspected to play a role in human longevity. Polymorphisms located near *APOE* and *FOXO3A* were found to be consistently associated with extreme longevity[5–7]; studies also yielded preliminary evidence for dozens of other candidate genes[8]. *APOE* was initially investigated because its ɛ4 allele was known to increase the risk of Alzheimer’s disease and coronary artery disease[5,9], while *FOXO3* was tested because its homologs influence lifespan in *C*. *elegans*, *Drosophila* and mice[10].

Beyond candidate gene studies, genome-wide association studies (GWAS) have been performed on long-lived cohorts to search for new longevity loci. Several GWA studies have identified variants near *APOE/TOMM40* to be consistently associated with longevity at genome-wide significance (P < 5 x 10^−8^)[3,11–14]. A locus at chromosome 5q33.3 has been found to be associated with longevity at genome-wide significance in one study, but has not yet been replicated[13]. One barrier to searching for longevity loci in GWAS is that centenarians are rare (only 17.3 per 100,000 people in the US[15]) and assembling sizable cohorts is challenging.

The relationship between exceptional longevity and disease is complex. The *APOE/TOMM40* locus, which shows the only consistent genome-wide significant association for longevity, is also implicated in age-related diseases and traits such as late-onset Alzheimer’s disease and cardiovascular disease, as well as others[16–20]. Furthermore, recent work showed that subjects with familial longevity have fewer risk variants for high levels of LDL cholesterol than controls[21]. However, there is some evidence that centenarians harbor anti-aging polymorphisms that protect them from disease, in which case they may carry many disease variants at a rate similar to normal people, but be protected from their effects. This possibility has been supported by several studies that have compared the total number of disease variants in centenarians and normal controls. These studies used whole genome sequencing or GWAS analyses to show that the genomes of centenarians contain many risk variants for disease[22,23], and that the overall count of disease-associated variants does not significantly differ for centenarians versus controls[3,24]. While these findings imply that many disease variants may not be depleted in centenarians, there may be additional variants, like the one at *APOE*, that are enriched in disease and depleted in centenarians[25]. In order to discover these longevity variants, one could take advantage of the results of large GWA studies of disease to help narrow the search.

In this paper, we develop a method to apply knowledge from disease and search directly for any genetic variants that predispose to age-related disease and are depleted in long-lived persons compared to controls. This analytic approach can amplify the statistical signals in the genetic data from small centenarian cohorts by taking advantage of information about SNPs already known to be associated with disease. First, we assembled a large collection of GWA data for age-related diseases and traits. We showed that several diseases, such as coronary artery disease and Alzheimer’s disease, exhibit significant genetic overlap with longevity. These results indicate that, among the set of SNPs associated with that disease, some are also depleted in centenarians. Next, we developed a new method called informed GWAS (iGWAS), which is a genome-wide statistical test for association with longevity that incorporates knowledge from disease. With our approach, a strong association signal in disease can boost a weak association for longevity to statistical significance. We applied our method by using data from 14 meta-analyses of disease in order to inform about longevity association in two long-lived cohorts. The method identified eight independent genetic loci to significantly associate with exceptional longevity, including five novel ones. We tested lead SNPs in independent long-lived cohorts, and replicated associations with longevity for two previously-known loci (*TOMM40/APOE* and *CDKN2B/ANRIL*) as well as two new loci (*ABO* and *SH2B3/ATXN2*). One additional locus (HLA) also showed some evidence of replication.

## Results

### Genetic variants associated with disease are more likely to associate with longevity

Some genetic variants that confer protection from disease could also associate with increased lifespan, and might show enrichment in centenarians and other long-lived populations. Previous studies used genetic risk scores for diseases, and failed to see a difference between centenarians and control groups, so not all, or even many, disease-associated variants will show this pattern[3,24]. However, there may be a few disease-associated SNPs that, like *APOE*, are depleted in centenarians; we computed the genetic overlap between longevity and disease to test this idea. We began by collecting summary statistics for 24 meta-analyses derived from 550 genome-wide association studies and subject cohorts available in the public domain or from consortia (Table 1). Of these, 21 meta-analyses involve disease (case/control) or a disease-related trait (quantitative) that are known to impact mortality to varying degrees[26–28]. In addition, we included three control meta-analyses involving a disease or trait that is not associated with age-related mortality. In total, our collection contains genome-wide SNP data involving > 1,150,000 disease cases and trait measurements (Table 1).

**Table 1:** Published GWAS meta-analyses for 24 diseases and traits.

**Disease**	**Acronym**	**# Cases**	**Reference**
Age-related macular degeneration	AMD	17,000	[17]
Type 2 diabetes	DIA	34,000	[68]
Rheumatoid arthritis	ART	5,500	[69]
Chronic kidney disease	CKD	5,807	[70]
Late-onset Alzheimer’s disease^a^	LOAD	8,000	[29]
Coronary artery disease	CVD	22,233	[71]
Pancreatic cancer	PANC	3,800	[72]
Lung cancer	LUNG	14,900	[73]
**Trait**	**Acronym**	**# Samples**	**Reference**
Bone density	BMD	32,961	[74]
High-density lipoprotein^b^	HDL	100,184	[19]
Low-density lipoprotein^b^	LDL	100,184	[19]
Triglycerides^b^	TRIG	100,184	[19]
Total cholesterol	TC	100,184	[19]
Body mass index	BMI	123,865	[75]
Waist-hip ratio^b^	WHR	123,865	[75]
Systolic blood pressure^a^ ^,^ ^b^	SBP	69,395	[76]
Diastolic blood pressure^a^	DBP	69,395	[76]
Beta-cell function^b^	HOMAB	46,186	[77]
Insulin resistance^b^	HOMAIR	46,186	[77]
Fasting insulin	INS	46,186	[77]
Adiponectin	ADIP	49,981	[78]
**Control**	**Acronym**	**# Samples**	**Reference**
Schizophrenia	SCZ	9394 cases	[79]
Major Depressive Disorder	MDD	9240 cases	[80]
Attention Deficit Hyperactivity Disorder	ADHD	2,064 trios	[81]

^a^Direction of effect was unavailable for these diseases and traits

^b^These traits were excluded from the cross-disease analysis

**Fig 1:**
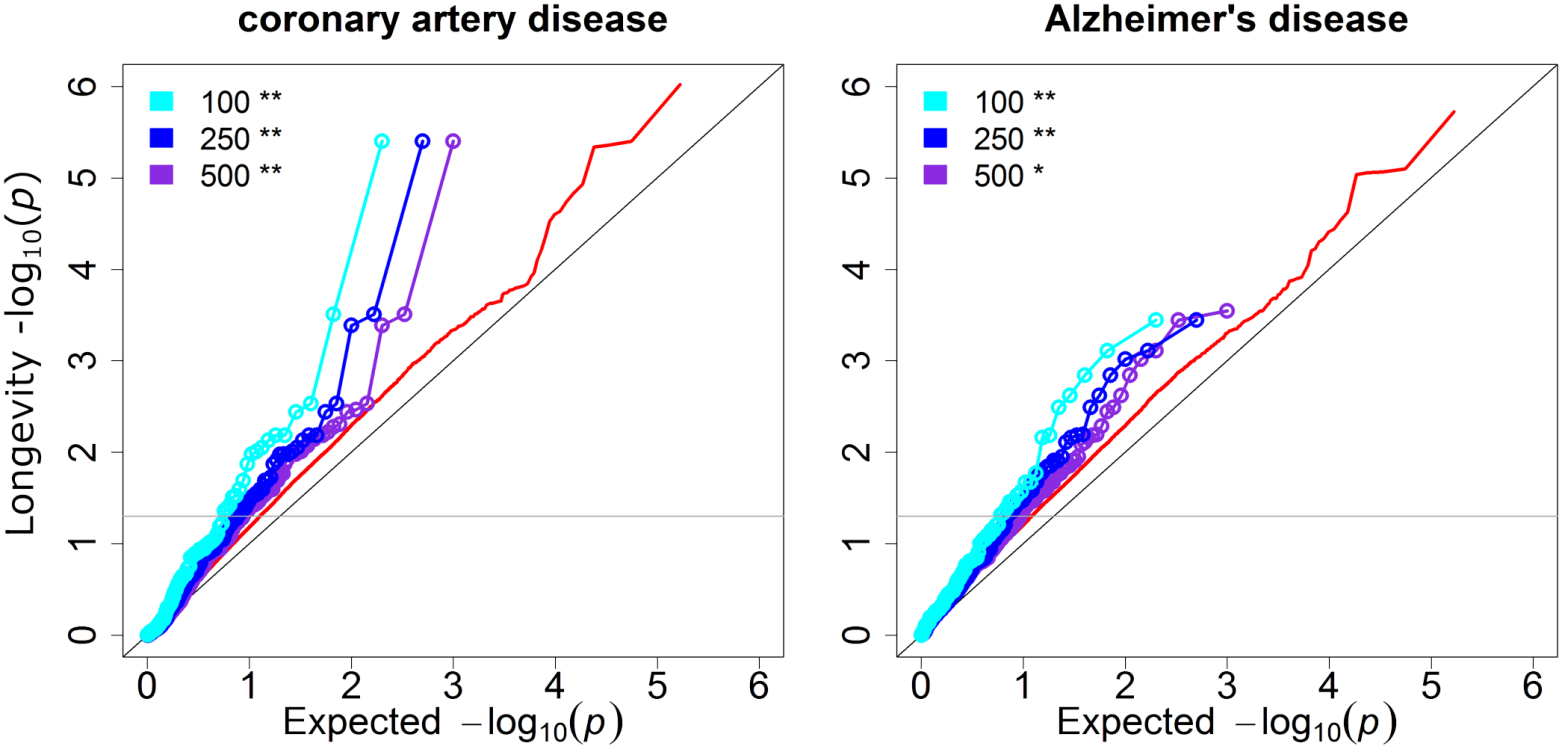
Disease GWAS show substantial genetic overlap with longevity. Shown are results for coronary artery disease and Alzheimer’s disease. The y axis is the observed P values for longevity[3], and the x axis is the expected P values under the null hypothesis that the disease is independent of longevity. The cyan, blue and purple lines show the P values for longevity of the top 100, 250, and 500 disease SNPs from independent genetic loci, respectively. Red lines show the background distribution of longevity P values for all independent genetic loci tested in both the longevity and disease GWAS. The grey horizontal line corresponds to the threshold for nominal significance (P< = 0.05) for longevity. Significance of enrichment was determined with the hypergeometric test. (*) P < 0.05, (**) P < 0.005. Q-Q plots for other diseases and traits are shown in S1 and S2 Figs.

We initially tested whether the set of genetic loci showing the strongest association with a disease is enriched for showing an association with longevity. For this test, we used summary statistics from the New England Centenarian Study (NECS), which is the largest centenarian GWAS to date with 801 cases[3]. For each of the 24 meta-analyses, we constructed a QQ plot that selects the top independent disease-associated loci, and displays their P values in the centenarian study (see Materials and Methods; Fig 1, S1 and S2 Figs). In each QQ plot, we make a list of the top ranking SNPs from a disease study, and plot their P values for longevity versus the P values that would be expected by chance. In this analysis, we used one-sided P values for the longevity data to test directly whether the allele enriched in disease is depleted in longevity (see Materials and Methods). For 3 of the 24 datasets, the summary statistics did not include direction of effect in which case we used two sided P values (Table 1). If there is no genetic overlap between the disease and longevity, then the top disease SNPs will follow the control line expected for a random distribution. If some of the top SNPs for disease also show an association with longevity, then the curve will be shifted to the left from the control line(s). To evaluate the significance of the genetic overlap of each disease and trait with longevity, we compare the number of top independent disease SNPs that are associated at P < 0.05 with longevity to the number expected by chance (Materials and Methods; P values and fold enrichments are given in S1 Table).

We found that 9 of 21 disease-related GWAS show significant genetic overlap with longevity (Fig 1, S1 Fig; S1 Table). For example, the QQ plots for coronary artery disease and Alzheimer’s disease show that the curves formed by the top disease SNPs are shifted left from the lines expected by a random distribution, indicating that some of the SNPs in the list are associated with longevity (Fig 1). The middle blue line shows the top 250 independent loci from the disease GWAS[29], the black line is a control showing uniformly distributed P values, and the red line is a control showing the P values for all loci tested (we selected the best SNP from each LD block in our pruning procedure for independent SNPs; see Materials and Methods). In the QQ plot, the blue line is shifted far to the left from both the red and black lines for both coronary artery disease and Alzheimer's disease, indicating that several SNPs with low P values for these diseases have low P values for longevity. In coronary artery disease, 13.2% of the top 250 disease SNPs are associated with longevity at nominal significance, which is more than the 7.9% that would be expected by chance from the background distribution of all independent SNPs represented by the red line (1.7 fold enrichment; P < 0.005). Likewise, for Alzheimer’s disease, 14.4% of the top 250 disease SNPs are nominally significant for longevity, which is more than the 8.4% that would be expected by chance (1.7 fold enrichment; P < 0.005). Besides the top 250 SNPs, the top 100 and 500 SNPs (cyan and purple lines, respectively) for either coronary artery disease or Alzheimer's disease show similar enrichment in the centenarian GWAS.

This same pattern of enrichment is repeated for other diseases and traits. In addition to coronary artery disease and Alzheimer’s disease, the diseases and traits showing a genetic overlap with extreme longevity were chronic kidney disease, diastolic and systolic blood pressure, three cholesterol traits (triglycerides, total cholesterol, Low Density Lipoprotein) and bone mineral density (Fig 1, S1 Fig; S1 Table).

The remaining 12 disease-related GWA meta-analyses and three control meta-analyses showed no genetic overlap with longevity (S2 Fig). This could mean either that these diseases are not relevant for exceptional longevity, or that we are not powered to detect a relationship with the datasets we used.

For every disease that shows an overlap with longevity, this analysis shows a general enrichment of disease SNPs in the centenarian GWAS but it does not identify which specific SNPs are most associated with extreme longevity. The particular SNPs that drive the genetic overlap in the QQ plots will be sensitive to the choice of threshold (e.g. whether the top 100 or the top 500 disease SNPs are examined), and there is no clear procedure for choosing the best threshold. Furthermore, it seems desirable to treat SNPs highly significant for disease differently from SNPs only moderately associated with disease. Finally, when a threshold is used in a candidate approach, we are no longer performing a genome-wide test because we ignore all SNPs that do not show a powerful association with disease. To more fully address these three issues, we developed a new method described below termed informed GWAS.

### A novel method to leverage knowledge from disease to identify longevity loci: informed GWAS (iGWAS)

Previously, a standard genome-wide analysis of the New England Centenarian Study dataset yielded one genome-wide significant locus, *APOE*, as well as 281 candidate loci that showed enrichment when combined into a genetic signature of exceptional longevity. The QQ plots in Fig 1 and S1 Fig show that there are additional genetic signals in the centenarian GWAS when the centenarian data are analyzed with the help of large GWA studies of disease. Here, we describe a new algorithm called informed GWAS (iGWAS) which leverages information from disease to inform GWA studies of longevity. The intuitive idea is that certain diseases may increase your mortality rate, and hence SNPs that increase your chance for those diseases may also decrease your chance to become a centenarian. SNPs with a large risk effect for a particular disease would be expected to have a correspondingly stronger effect on longevity than SNPs with a weak effect on that disease. This suggests a strategy to assign weights to SNPs in a GWAS for longevity based on their risk for disease (a P value weighting strategy), a method described more fully in Dobriban et al, 2015[82]. There are many ways to assign weights to different disease SNPs to be subsequently used in a longevity analysis. For instance, the top disease SNPs could be given an equal weight (i.e. candidate gene approach).

iGWAS is a new method that optimizes the results from the P value weighting strategy[30]. P value weighting yields a genome-wide test that can increase the statistical power to detect associations for certain SNPs (those for which we have some prior knowledge, which is reflected in the weights assigned to each P value), at the cost of reduced power for other SNPs. In our application, weighting can improve our power to detect longevity associations for those SNPs previously implicated in disease, but can miss other types of longevity SNPs that are not associated with disease. In general, the task in P value weighting is to derive a set of weights (*w*_*s*_) to be applied to P values (*P*_*s*_) of a target GWAS or other high-throughput study[31–33]. Weighted P values (*P*_*s*_/*w*_*s*_) are used instead of P values (*P*_*s*_) used in multiple hypothesis testing. The SNPs with large weights from disease GWAS yield smaller weighted P values, and the associated SNPs are more likely to be declared significant. Most of the previous applications of P value weighting assigned weights using simple heuristic formulas[31,32]; iGWAS relies on a principled approach where weights are chosen such that they maximize the expected number of loci discovered (S1 Text). P value weighting has not been previously applied to inform one GWAS using knowledge from another GWAS of a different phenotype.

We assume that the test statistics from the longevity GWAS, i.e. the Z scores for each SNP *i*’s association with longevity (normalized by sample size), are distributed as ~*N*(*μ*_*i*_,1). The key assumption behind iGWAS is that the results of disease GWAS can be used to construct an estimate of the longevity GWAS vector *μ*_*i*_. This strategy can work to identify new SNPs so long as there are some SNPs that influence both disease risk and one’s chances of exceptional longevity. We can treat the observed test statistics *η*_*i*_ in the disease GWAS as an imprecise guess of the test statistics for the longevity GWAS. Since disease and longevity are not the same phenotype, this guess will be imprecise. We model this by assuming that, from prior knowledge from disease GWAS, we have a prior distribution for *μ*_*i*_, given by *μ*_i_*~*N*(*η*_*i*_,*σ* ^2^)(See S1 Text). The *η*_*i*_ are given in disease GWAS, and we choose the parameter *σ* to reflect our uncertainty in our estimate of each *μ*_*i*_. The main source of uncertainty in the current application stems from the fact that longevity and disease are different phenotypes, and not all strong disease SNPs will be strong longevity SNPs (Methods).

Using *η*_*i*_ derived from the disease GWAS we build weights *w*_*i*_ to be applied to the P values of each of *S* SNPs in the longevity GWAS to yield weighted P values *P*_*i*_ */w*_*i*_. In weighted multiple testing. The intuition here is that weighting can allow us to take advantage of knowledge from disease by increasing our power to detect an association with longevity for those SNPs that strongly associate with disease. Since the weights *w*_*i*_ must average to 1, this increase in power for disease SNPs comes at a cost of a decrease in power for the rest of the genome. There are many possible choices of the weight vector w; here we derive an optimal vector of weights that maximizes the expected number of significant SNPs in the longevity GWAS, given the assumptions about how the disease test statistics are related to the longevity test statistics. Let the number of rejections be given by $R(w)\;=\;\sum_{i\;=\;1}^{S}I(P_{i}\leq qw_{i})$, where S is the number of SNPs being tested, and q is the threshold for rejection. Then our task is to find the weight vector that will maximize the expected number of significant rejections, which can be formulated as an optimization problem:

$$\underset{w}{\text{max}}\;E_{\mu }E_{T}R(w)$$

$$\text{subject to }w_{i}\geq 0,\bar{w}\;=\;1$$

The constraints on *w* guarantee that the False Discovery Rate is controlled using the weighted P values[30]. Further statistical details and a derivation of the weights *w*_*i*_ that solve this optimization problem are given in S1 Text.

After calculating the iGWAS weights, they can be applied to the raw P value of the target longevity GWAS to yield weighted P values *P*_*i*_ */w*_*i*_.The weighted P values can then be adjusted for multiple testing with the Benjamini Hochberg step-up procedure. If the weights *w*_*i*_ are nonnegative and average to 1 and the P values are independent, then the false discovery rate is controlled at the desired level [33]. Here, we apply our method to increase power in longevity GWAS by using prior knowledge from disease, but we anticipate that iGWAS will find wide application in other GWA studies.

Other methods have been proposed to take advantage of prior knowledge in a GWAS. The simplest such strategy is a candidate approach, where only a subset of all SNPs are tested for their association to longevity, e.g. the top N SNPs that are known to associate with a disease. Another genome-wide method that takes advantage of prior knowledge from GWAS results is described as the conditional FDR method [34,35]. This method controls the conditional False Discovery Rate, defined as the posterior probability that a SNP is null for the target phenotype given that the P values for both the target and prior knowledge phenotypes are as small as or smaller than the observed P values. The conditional FDR method is a Bayesian procedure that does not guarantee control of the commonly used frequentist false discovery rate. P value weighting, on the other hand, provides a flexible framework for incorporating prior knowledge that can control the usual frequentist FDR at the desired level [30], and iGWAS provides a rational strategy for choosing a vector of weights that can increase the number of discoveries and make full use of the proxy phenotype.

### Eight loci are significantly associated with extreme longevity

**Fig 2:**
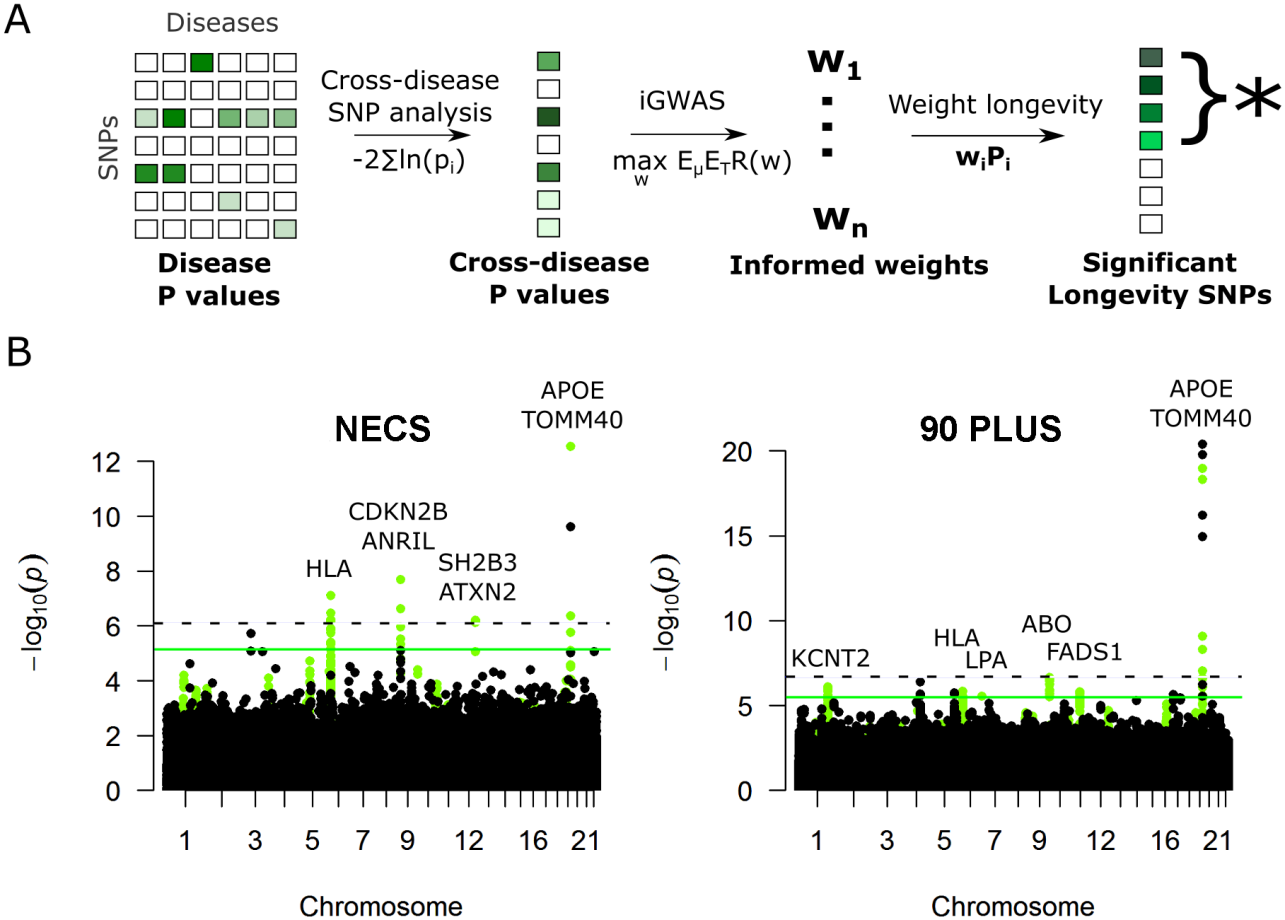
Informed GWAS implicates 8 loci in longevity. **A.** Shown is a basic schematic of our method for deriving weights. First, we perform a cross-disease analysis across all meta-analyses of disease GWA studies. Next, we use the results of the cross-disease analysis to derive weights for the longevity GWAS. Finally, we weight the P values from the longevity studies, and identify significant SNPs. **B**. Weighting boosts new loci to significance. Shown are Manhattan plots of all SNPs before (in black) and after (green) adjusting their P values with disease-informed weights. The black and green lines show the FDR < 10% cutoff in the unweighted and weighted GWAS, respectively. Without weights, only the *APOE* locus is significant in NECS and 90PLUS; after weighting, 7 more loci become statistically significant at FDR < 10%. The x axis shows SNPs according to their chromosomal positions.

**Fig 3:**
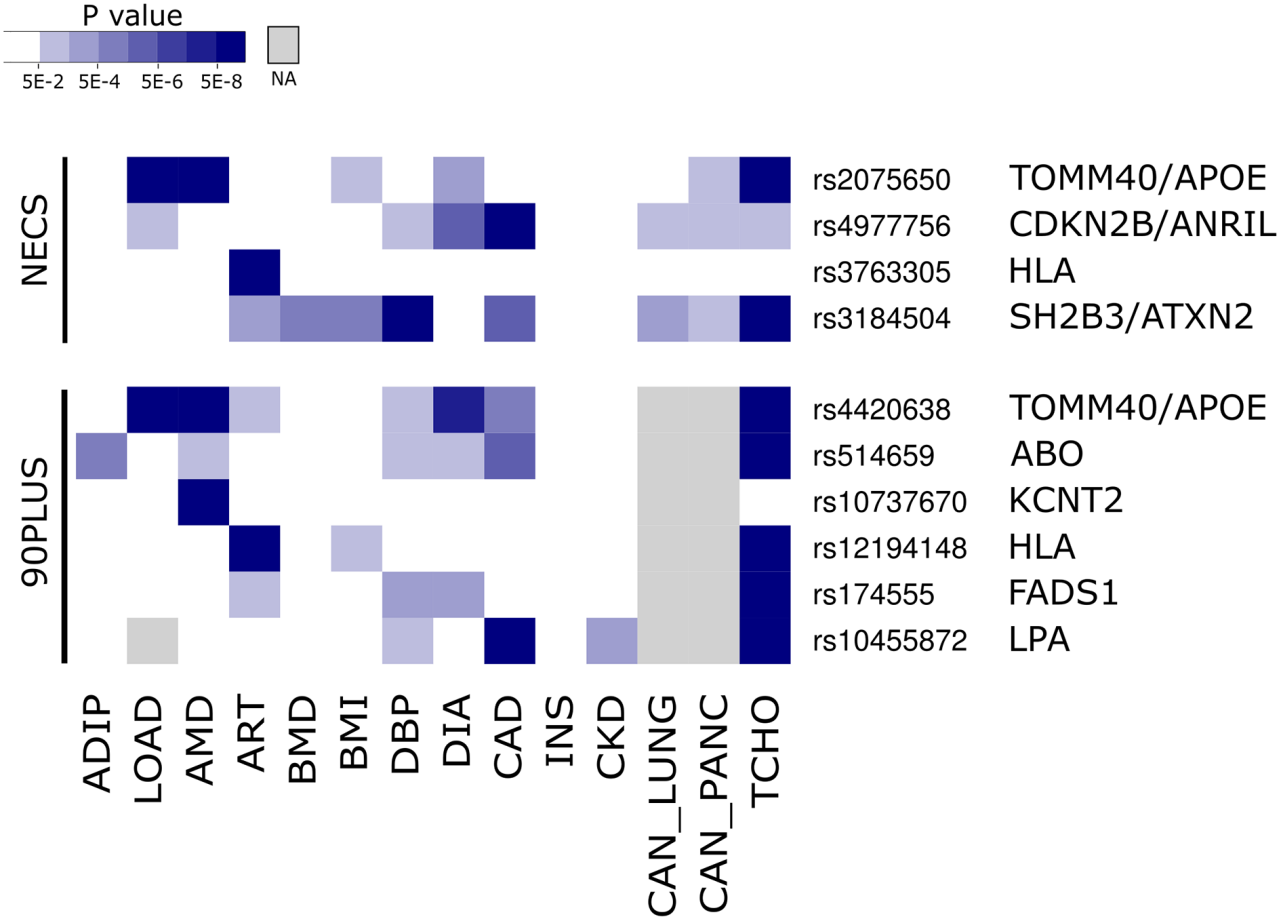
Nominal associations with multiple diseases for longevity loci. Shown are raw P values for each lead longevity SNP in each of the 14 meta-analyses of disease GWA studies that contributed to the cross-disease analysis used by iGWAS. Colors of blue indicate P values in the disease GWAS. The darkest blue corresponds to genome-wide significance. ADIP, adiponectin levels; LOAD, late-onset Alzheimer's disease; AMD, age-related macular degeneration; ART, rheumatoid arthritis; BMD, bone mineral density; BMI, body mass index; DBP, diastolic blood pressure; DIA, type 2 diabetes; CAD, coronary artery disease; INS, fasting insulin levels; CKD, chronic kidney disease; CAN_LUNG, lung cancer; CAN_PANC, pancreatic cancer; TCHO, total cholesterol.

We used genome-scale data from two recent GWA studies of human longevity: the New England Centenarian Study (NECS) includes 801 centenarians and 914 controls[3], and the 90PLUS study includes 5406 elderly over age 90 and 15112 controls[13]. We applied iGWAS to find variants associated with extreme longevity (Fig 2A). First, we calculated a score for each SNP that reflects its aggregate involvement in disease; specifically, for each SNP we combined its P values across diseases and traits into a single P value using Fisher's method. In this way, a SNP may have a low P value in our cross-disease analysis if it is strongly implicated in one disease, or alternatively if it shows a pattern of low P values across multiple distinct diseases (Fig 3). To minimize over-representation from highly-correlated traits (e.g. four related measurements of cholesterol levels), we eliminated 7 disease or trait categories that were clearly redundant, resulting in a total of 14 diseases and traits (Table 1). Next, we converted cross-disease analysis P values into Z scores, and used the Z scores as input to iGWAS for two longevity GWAS (NECS and 90PLUS).

Using iGWAS at a false discovery rate of 10%, we discovered eight independent loci that showed significant associations with longevity (Fig 2B). These loci are represented by 92 unique significant SNPs (S2 Table). Using a standard GWAS approach, only the *APOE* locus would have been significant at FDR < 10% (Fig 2B). Of the eight loci from iGWAS, two linkage disequilibrium (LD) blocks showed longevity associations in both studies. The first of these includes the *APOE/TOMM40* locus (Fig 2B). The second LD block contains the *HLA* region on chromosome 6. Separate lead SNPs from this LD block were associated with extreme longevity using iGWAS for both longevity cohorts; specifically, rs3763305 showed an association in NECS and rs12194148 showed an association in 90PLUS (Fig 2B). Because these two lead SNPs fall in the same LD block, the independent iGWAS results may be converging on the same genetic signal. Two additional loci were significantly associated with longevity only in the NECS study and four additional loci were found only in the 90PLUS study (Fig 2B, Table 2 and S2 Table).

**Table 2:** Regularized weights informed by disease implicate 8 loci in human longevity.

**Source**	**SNP**	**Candidate Gene(s)** ^a^	**Raw P** ^b^	**Odds Ratio (CI)** ^c^	**Weighted P** ^d^	**FDR** ^e^	**Alleles** ^f^
NECS	rs2075650	*TOMM40/APOE*	2.36E-10	.48(.35-.67)	3.68E-13	8.98E-08	**A**/G
NECS	rs4977756	*CDKN2B/ANRIL*	7.97E-06	.73(.60-.89)	2.46E-08	2.11E-03	A/**G**
NECS	rs3763305	*HLA*	6.27E-05	.53(.34-.82)	9.78E-08	4.84E-03	G/**A**
NECS	rs3184504	*SH2B3/ATXN2*	5.05E-04	.79(.65-.95)	7.88E-07	1.92E-02	**G**/A
**Source**	**SNP**	**Candidate Gene(s)** ^a^	**Raw P** ^b^	**Odds Ratio (CI)** ^c^ ^,^ ^g^	**Weighted P** ^d^	**FDR** ^e^	**Alleles** ^f^
90PLUS	rs4420638	*TOMM40/APOE*	4.09E-21	.64(.59-.70)	5.73E-24	1.40E-17	**A**/G
90PLUS	rs514659	*ABO*	0.000179	.89(.84-.95)	2.51E-07	4.53E-2	**A**/C
90PLUS	rs10737670	*KCNT2*	0.000923	.90(.85-.96)	1.29E-06	7.84E-2	A/**G**
90PLUS	rs12194148	*HLA*	0.001118	.91(.86-.96)	1.57E-06	7.84E-2	**G**/T
90PLUS	rs174555	*FADS1*	0.001113	.91(.85-.96)	1.56E-06	7.84E-2	T/**C**
90PLUS	rs10455872	*LPA*	0.002085	.84(.75-.94)	2.72E-06	9.85E-2	**A**/G

^a^Candidate gene for longevity

^b^P value using longevity data alone

^c^Allelic odds ratio (95% confidence interval)

^d^P value using iGWAS

^e^False Discovery Rate of iGWAS

^f^Major/minor alleles. Protective allele in bold.

^g^For SNPs in 90PLUS (except rs4420638), odds ratios are not available from summary data and are estimated using allele frequencies from 1000 Genomes EUR.

### Replication of longevity associations

To replicate our findings, we obtained data from three additional long-lived cohorts: centenarians from Southern Italy (containing 410 centenarians and 553 controls; SICS)[36], from Northern Italy (226 centenarians and 220 controls; NICS), and centenarians of Ashkenazi Jewish descent (474 centenarians and 799 controls; LGP). SNPs that were discovered in the NECS or the 90PLUS studies were replicated in the remaining data sets. We tested seven of our eight lead SNPs for replication. For the eighth SNP (rs10455872 in *LPA*), neither the SNP itself nor any strongly linked SNP was available in any replication cohort.

We tested each of the seven SNPs for an effect in the same direction as in the discovery sample. P values for each of the three replication cohorts were combined into a meta-analysis P value by Stouffer’s method (Table 3). After controlling the false discovery rate at 5%, four loci replicate in our validation cohorts; these loci are defined by lead SNPs rs2075650/rs4420638 (*APOE/TOMM40*, chr. 19), rs4977756 (*CDKN2A/ANRIL*, chr. 9), rs3184504 (*SH2B3/ATXN2*, chr. 12) and rs514659 (*ABO*, chr. 9)(Table 3). A fifth LD block, containing the *HLA-DR* region, was identified by iGWAS in both the NECS and 90PLUS studies, although the lead SNPs in each study were different. In summary, five of seven loci discovered to be associated with extreme longevity by iGWAS show some evidence of replication in follow-up studies.

**Table 3:** Validation results for candidate longevity loci.

**Source**	**SNP**	**Candidate Gene(s)**	**Protective allele**	**P 90PLUS**	**P SICS**	**P NICS**	**P LGP**	**Combined P** ^a^
NECS	rs2075650	*TOMM40/APOE*	A	3.06E-17	3.12E-02			**2.40E-13**
NECS	rs4977756	*CDKN2B/ANRIL*	G	1.99E-02	1.18E-01	0.55	7.75E-03	**2.82E-03**
NECS	rs3763305	*HLA*	A	9.08E-02			4.97E-01	**ns**
NECS	rs3184504	*SH2B3/ATXN2*	G	1.01E-01	7.75E-02^b^		8.53E-02	**9.41E-03**
**Source**	**SNP**	**Candidate Gene(s)**	**Protective allele**	**P NECS**	**P SICS**		**P LGP**	**Combined P** ^a^
90PLUS	rs4420638	*TOMM40/APOE*	A					
90PLUS	rs514659	*ABO*	A	1.60E-02^b^ ^,^ ^c^	3.24E-01^b^		4.48E-02	**6.55E-03**
90PLUS	rs10737670	*KCNT2*	G		4.21E-02^b^		7.87E-01	**ns**
90PLUS	rs12194148	*HLA*	G		3.98E-01^b^			**ns**
90PLUS	rs174555	*FADS1*	C	4.31E-01^b^	1.80E-01^b^		4.33E-01	**ns**
90PLUS	rs10455872	*LPA*	A					

All P values reported are effect-concordant, one-sided replication P values. A grey rectangle indicates that no SNP or proxy data was available. “ns” = not significant.

^a^Combined P across all replication cohorts calculated using Stouffer’s test (Materials and Methods)

^b^Lead SNP missing from data set; best proxy SNP used instead (with R^2^ > 0.8, D’ = 1; see S4 Table).

^c^rs514659: the proxy SNP we use here is nominally significant in NECS (Table 3), but the imputed P value is not (S5 Table; Materials and Methods).

**Fig 4:**
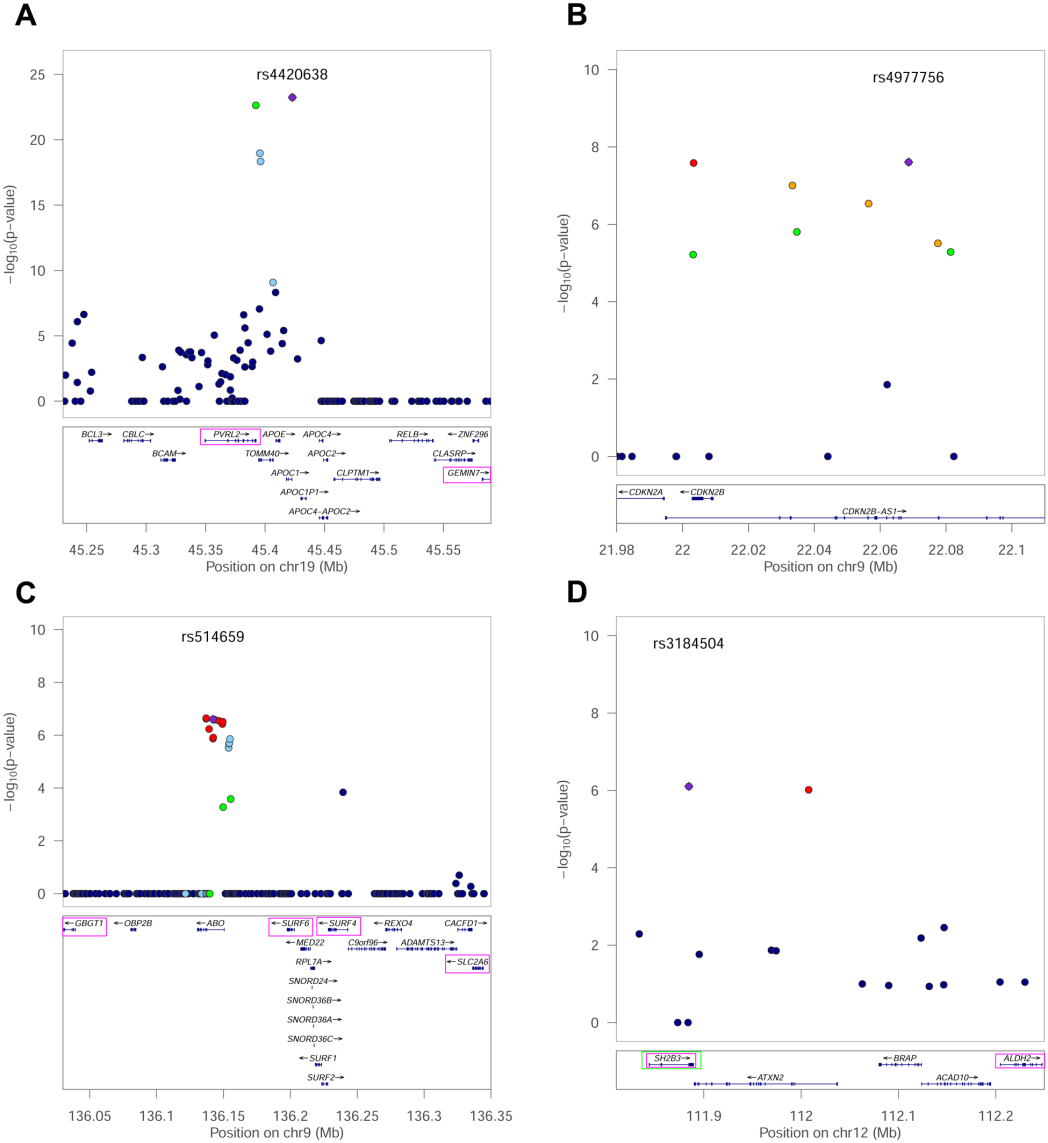
Regional plots for four longevity-associated SNPs. The y axis shows weighted P values and the x axis indicates position of the SNPs. Color of the SNP indicates linkage to the lead SNP (purple). Green boxes indicate genes harboring missense SNPs in LD with candidate longevity SNPs, and magenta boxes indicate genes whose expression is associated with a candidate longevity SNP (eQTL). A. Data for rs4420638 in APE/TOMM40. B. Data for rs497756 in CDKN2B. C. Data for rs514659 in ABO. D. Data for rs3184504 in SH2B3.

These five loci associated with extreme longevity contain one or more known aging-related candidate genes with functions that could be mediating their effect on disease and longevity. However, since the lead SNPs are contained in large linkage disequilibrium blocks that often contain many genes, it could be that the longevity effect is due to linked mutations that affect other genes in the LD block (Fig 4, S2 Fig). For each of the lead SNPs from iGWAS, we searched for linked SNPs that alter the protein coding sequence (i.e. missense SNPs) or gene expression in peripheral blood (S3 Table)[37].

The first locus is on chromosome 19, and contains *TOMM40* and *APOE*, which were previously shown to associate with extreme longevity at genome-wide significance (Fig 4A)[3,11–14]. The lead SNPs identified by iGWAS (rs4420638 and rs2075650) have previously been shown to have associations with Alzheimer's disease, age-related macular degeneration, coronary artery disease, and total cholesterol levels that are genome-wide significant[38]. These lead SNPs are not closely linked (R^2^ < 0.5) to the two SNPs that define the pathogenic ApoE4 allele (rs429358 and rs7412). Neither of these ApoE4 SNPs were queried in the iGWAS longevity analyses, but past conditional analyses have found that the genetic signal at rs2075650 is dependent on the polymorphisms that define the E4 haplotype[11]. In the iGWAS analysis, the rs4420638 allele enriched in centenarians (A) shows a protective effect for Alzheimer’s disease, total cholesterol, and pancreatic cancer. Searching for expression quantitative trait loci (eQTLs) in the region showed SNPs that affect expression in two genes (PVRL2 and GEMIN7). If the longevity effect is not due to *TOMM40* or *APOE* itself, then these two genes are candidates to explain the association with extreme longevity. rs2075650 explains 1.2% of the variance in the longevity phenotype.

The second locus is located at chromosomal position 9p21 (Fig 4B). This locus has been previously implicated in longevity; it was included in the 281-SNP signature of longevity derived from NECS data[3], and found to replicate in additional long-lived cohorts in a follow-up paper (at FDR <5%)[39]. Additionally, two preliminary candidate studies have tested the 9p21 SNP rs1333049 for differences in centenarians, finding promising though inconclusive evidence[40,41]. SNPs from this region have previously been found to be associated with a surprising diversity of age-related diseases, including cardiovascular disease, type 2 diabetes, intracranial aneurisms, amyotrophic lateral sclerosis and several cancers[38,42,43]. For the cardiovascular disease GWAS, this locus shows the strongest association of any locus in the genome, with each copy of the risk allele increasing one’s risk of disease by 20–30%[44]. In the iGWAS analysis, the centenarian-enriched allele of the lead SNP (rs4977756, G) is protective for coronary artery disease and diabetes. This locus contains two protein-coding genes (*p15/CDKN2A* and *p16/CDKN2B*) that inhibit the cell cycle and promote cell senescence[45]. The LD block also contains a long antisense RNA (*CDKN2B-AS1/ANRIL*) that may regulate the expression of *CDKN2A* and *CDKN2B*[46]. The functions of these genes support a role for cell senescence in extreme longevity.

The third locus contains the *ABO* blood group gene (Fig 4C). The lead SNP (rs514659) is strongly linked to the SNP that defines the most common allele (O1) responsible for the O blood group (rs8176719; R^2^ = 0.97); O1 is a frameshift mutation in the *ABO* gene causing early termination. Our results show that centenarians are more likely to have the O blood group than controls. People with blood type O have been reported to be protected from coronary heart disease[47], cancer[48], and have lower cholesterol levels[19,49]. In the iGWAS analysis, this SNP showed a nominal association with six diseases/traits, consistent with the idea that the O blood group associates with many protective effects (Fig 3).

The fourth locus contains *SH2B3* and *ATXN2*, which are attractive candidate genes for extreme longevity (Fig 4B). Variation in this locus has been associated with a wide variety of diseases, including rheumatoid arthritis, type 2 diabetes, coronary artery disease, blood pressure and cholesterol levels[38]. In the iGWAS analysis, the centenarian allele of the lead SNP (rs3184504, G) is protective for lung and pancreatic cancer, coronary artery disease, rheumatoid arthritis, diastolic blood pressure, and bone mineral density. *SH2B3* encodes a signaling protein and loss-of-function mutations in its ortholog (*Lnk*) in *Drosophila* results in extended lifespan[50]. The lead SNP from the iGWAS analysis (rs3184504) is a missense SNP in *SH2B3* (R262W). The lead SNP is also associated with expression variation for *SH2B3* as well as another gene in the same LD block *ALDH2*. The protective allele (G) in human *SH2B3* is associated with lower expression of *SH2B3* in peripheral blood[37], consistent with the loss-of-function results for lifespan extension in *Drosophila Lnk*. *ATXN2* is a poly-glutamine repeat containing protein involved in many neurodegenerative disorders such as amyotrophic lateral sclerosis (ALS) and Spinocerebellar Ataxia type 2 (SCA2)[51].

The fifth locus contains the *HLA-DR* and *HLA-DQ* genes involved in histocompatibility, as well as about 100 additional genes in a large LD block spanning about three megabases on chromosome 6 (S3 Fig). The *HLA-DR* and *HLA-DQ* genes are highly polymorphic and have been associated with over 40 diseases, including narcolepsy, rheumatoid arthritis, multiple sclerosis and myasthenia gravis[38]. In the iGWAS analysis, the centenarian allele of rs12194148 (G) is associated with lower cholesterol levels and protection from rheumatoid arthritis. One of the lead SNPs from this locus, rs3763305, was previously included in the 281-SNP signature of longevity, and found to replicate in additional long-lived cohorts in a follow-up paper (FDR <5%)[39].

## Discussion

We have shown that one of the genetic mechanisms for extreme longevity involves the avoidance of certain risk alleles that predispose to common diseases, including coronary artery disease, Alzheimer’s disease, high cholesterol and chronic kidney disease. For these diseases, QQ plots show that some of the top disease SNPs have increased likelihood of having low P values for longevity, with the risk allele for disease showing depletion in centenarians.

Previously, Beekman et al.[24] and Sebastiani et al. used sets of disease SNPs to construct genetic scores and found no significant difference in the number of disease alleles in centenarians vs. controls [3,23,52]. Several factors could contribute to the difference between the results from the QQ plot and from several genetic score analyses. There may be some disease variants that are enriched in centenarians as well as others that are depleted; when the two types of disease variants are combined in a genetic score, their effects are cancelled giving rise to similar genetic scores in centenarians and controls. Also, if most disease variants are carried at a similar proportion in centenarians and controls, very large cohorts may be required to detect a difference in a genetic score.

By applying a new statistical method, iGWAS, we have identified lead SNPs for exceptional longevity in eight loci. Four loci were replicated and one locus was partially replicated in independent centenarian cohorts. These five loci contain genes that are attractive candidates for mediating the disease and longevity effects: 1) *TOMM40/APOE*, 2) *CDKN2B/ANRIL*, 3) *ABO*, 4) *SH2B3/ATXN2*, and 5) *HLA-DR* and *HLA-DQ*. Each of these loci suggests important mechanisms for extreme longevity; for instance, the *ANRIL/CDKN2B* locus suggests that cell senescence may play a role in extreme longevity. Future studies with larger cohorts of centenarians will be needed to validate the association of these loci with longevity at genome-wide significance levels.

One issue with the analysis of any centenarian study is the age of the control cohort. The allele frequency of SNPs as a function of age may not change in a simple monotonic fashion[53–55]. In particular, alleles for some SNPs show a U-shaped pattern such that young and very old people have similarly high allele frequencies, but 80 year olds have a lower level. In this case, choice of the control cohort would affect whether or not an allele is significantly different in centenarians.

iGWAS uses information from large amounts of genetic data on diseases to find one particular type of longevity SNP where, as with *APOE*, the disease allele is depleted in long-lived populations. A second genetic mechanism for extreme longevity involves protective effects in centenarians that buffer from the negative effects of other common disease risk alleles[53].

To find specific loci associated with extreme longevity, we developed an approach termed informed GWAS. The challenge was to find SNPs associated with longevity given a relatively small cohort size (e.g. 801 centenarians in the NECS cohort). iGWAS boosts the statistical power to find disease-associated SNPs in the small longevity GWA study by using information from large amounts of genetic data on diseases. iGWAS is a P value weighting framework that chooses principled (rather than heuristic) weights from the disease data.

Aging is the major risk factor for most diseases. Hence, for GWA studies of age-related diseases, any locus might be primarily identified because it is specifically and directly involved in that disease (i.e. a disease SNP). Another possibility is that the locus might be indirectly involved in that disease, by affecting a process underlying multiple age-related diseases (i.e. an aging SNP). One way to begin to distinguish a variant that influences a general underlying mechanism of aging from one that reduces risk for a specific disease is that the former would be expected to show an association across many distinct diseases. Intriguingly, many of the SNPs found by iGWAS showed an association for not one, but many diseases which seem to have distinct etiologies. Examples include the association of the *APOE/TOMM40* locus with pancreatic cancer and total cholesterol levels, the association of the *ABO* locus with macular degeneration and coronary artery disease, and the association of the *SH2B3/ATXN2* locus with diastolic blood pressure and rheumatoid arthritis. These may be part of a new class of SNPs that are not specific to any one disease, but instead are associated with a general mechanism (such as reduced rate of aging) that acts across multiple diseases.

Besides some disease SNPs that are depleted in centenarians, it is also plausible that centenarians carry a different genetic background than the normal population consisting of protective SNPs that predispose for extreme longevity. Future work will be required to reveal these additional types of loci for extreme longevity, such as whole genome sequencing studies of extremely long-lived people [22,23,56].

Beyond the study of human longevity, informed GWAS could be applied to other GWA studies, such as diseases or traits that show some co-morbidity or correlation. iGWAS could substantially boost statistical power in any GWAS of a target phenotype (e.g. a disease such as type 2 diabetes) by using larger GWAS of genetically-related conditions (e.g. body mass index, which is a risk factor for type 2 diabetes). iGWAS may aid discovery in any GWAS, where despite large sample sizes, many loci of modest effect remain undetected.

## Materials and Methods

### Disease and trait GWAS summary data

Genome-wide summary data from 24 diseases (case /control studies) and quantitative traits (e.g. cholesterol levels) were obtained from online web resources, or provided by the organizing consortia (Table 1). In each dataset, SNPs and genome-wide P values were provided; some studies also included further information such as effect size, risk allele, standard error, etc.

### Longevity discovery and replication cohorts

#### NECS

New England Centenarian Study[3]; based on 801 centenarian cases (age 95–119; median survival 104 years) and 914 controls, all Caucasian. Control subjects were selected to match the genetic background of cases, and included 241 NECS controls (spouses of centenarian offspring, children of parents who died at a mean age of 73), and 673 Illumina database controls with age range at blood draw between 0 and 75 years. We used two-sided genome-wide summary P values for 243,980 SNPs calculated under an allelic model. NECS was used as a discovery cohort, and as a replication cohort for SNPs discovered from the 90PLUS cohort.

#### 90PLUS

Meta-analysis incorporating 7330 cases aged 90 or older and 16121 younger controls (age 65 or less), all of European descent[13]. Genome-wide summary P values for over 2 million SNPs were provided as two-sided P values calculated from a fixed-effects meta-analysis. 90PLUS was used as a discovery cohort, and as a replication cohort for SNPs discovered from NECS data.

### Replication cohorts

#### SICS

Southern Italian Centenarian Study[36]; based on 410 centenarians (age 90–109) and 553 controls from Southern Italy. We used two-sided genome-wide summary P values for 280,890 SNPs calculated under an allelic model.

#### LGP

Longevity Genes Project[57]; included 474 centenarians (age 95–110) and 799 controls, all of Ashkenazi Jewish ancestry. We were provided with two-sided P values for candidate SNPs calculated using the Wald test.

#### NICS

Northern Italian Centenarian Study; based on 226 centenarians (mean age 102.3) and 220 controls from Northern Italy. Lead SNPs discovered in NECS were analyzed by Sequenom MASSArray or Real Time PCR. Results for rs3184504 and rs3763305 failed the test of Hardy-Weinberg equilibrium, likely due to the small size of the cohort. We were provided with two-sided P values calculated under an allelic model.

### Analyzing the genetic overlap of longevity and disease

For each of 21 disease-related meta-analyses, and 3 control meta-analyses, we determined whether SNPs that have small P values for disease also have small P values for longevity. First, we determined which SNPs were tested in both the longevity GWAS and the disease GWAS. Next, we pruned this set of SNPs to an independent list of so that no pair would be in linkage disequilibrium (LD > 0.2) using European samples from the 1000 Genomes Project[58] (EUR Phase I Integrated Release Version 3 Haplotypes). SNPs were ranked by their P value for longevity, then starting with the first SNP in the list, all later SNPs with LD > 0.2 were removed using the clump tool from plink[59]. Furthermore, we removed *APOE* (rs2075650) from the list (and anything in LD > 0.2) as it is already genome-wide significant in NECS. From the final set of 83,304 LD-thinned SNPs, we determined the top 100, 250 or 500 disease-linked loci for each disease. We then evaluated how many of these loci were nominally associated with longevity with P < 0.05. For 18 disease datasets and 3 control datasets where direction of effect was given, we converted the two-sided longevity P values to one-sided longevity P values preserving the direction of effect in disease to test directly whether disease risk alleles are depleted in longevity. For longevity and disease effect directions *e*_*long*_ and *e*_*dis*_ these are calculated as:

$$p_{one-sided}=\{\begin{array}{ll} p_{two-sided}/2 & if\;e_{long}=\;e_{dis}\\ \;1-\;p_{two-sided}/2 & \;if\;e_{long}\neq e_{dis} \end{array}$$

Thus, QQ plots for these diseases show only those SNPs where the risk alleles are depleted in centenarians with one-sided longevity P < 0.05 (Fig 1, S1 and S2 Figs). For three datasets where effect direction was unavailable (Table 1), we used two-sided P values.

We then applied the hypergeometric test to compare the number of nominally significant associations for disease SNPs to the number expected by chance (S1 Table).

### Implementing iGWAS

We developed iGWAS to use disease GWAS results as prior knowledge to derive a vector of weights for longevity GWAS (Results; S1 Text). We gathered information from meta-analyses of GWAS for different diseases and traits, and then analyzed the pooled data across all diseases to identify SNPs strongly implicated in one or more diseases. Although the QQ plots indicated that certain traits showed more overlap with longevity than other traits, we chose to evaluate all of the traits similarly in the GWAS analysis to avoid an unwanted bias. If we were to first search for overlaps, and then give higher weights to those traits in iGWAS, a false signal in the first QQ analysis would be inadvertently amplified in the second iGWAS analysis that might lead to a spurious result.

Genome-wide results of this cross-disease analysis were then applied to weight the longevity P values using iGWAS. From our initial set of 21 meta-analyses of disease-related GWAS, some involved related traits measured on the set same set of individuals (and were published in the same paper; see Table 1). For each of the four papers containing results for more than one related trait (e.g., one paper contained data on four cholesterol traits), we chose one trait to include in the meta-analysis. In total, we used 14 GWAS in the meta-analysis: Coronary artery disease, type 2 diabetes, Alzheimer’s disease, age-related macular degeneration, lung cancer, pancreatic cancer, rheumatoid arthritis, total cholesterol, insulin, body-mass index, bone mineral density, adiponectin levels, and chronic kidney disease (Table 1). For each SNP, P values from each of the 14 studies were combined using Fisher’s method (as implemented in the CRAN R package MADAM[60]). We used two-sided P values and did not take direction of effect into account (risk vs. protective alleles), as this information was unavailable for some disease datasets. For each SNP, the disease P values *p*_*i*_ are combined to give a test statistic *S*:

$$S\;=\;-2\text{log}\;\prod_{i\;=\;1}^{k}p_{i}$$

which follows a χ^2^ distribution with 2 *k* degrees of freedom. The Fisher meta-P values were then converted to Z scores and used to derive weights for the longevity study. Once specific SNPs had been identified by iGWAS, the direction of effect in the discovery GWAS (NECS or 90PLUS) was taken into account in the replication analysis.

We estimated the strength of association of each SNP with longevity (i.e. its level of significance) directly from the SNP’s known strength of association with disease. Let *N*_*L*_ and *N*_*D*_ be the sample sizes of the longevity and disease study, let *S* be the total number of SNPs, and for each SNP *s* let $Z_{D}^{s}$ be its Z score (standard score) for association with disease (from the meta analysis). Given $Z_{D}^{s}$, we can estimate the Z score for longevity as $Z_{L}^{s}\;=\;\sqrt{N_{L}/N_{D}}\;Z_{D}^{s}$. We then computed the optimal weights for longevity, setting $\eta_{S}\;=\;{|Z}_{L}^{S}|$, *σ* = 0.25, and *q = 1/S* (Results; S1 Text). Although *η*_*S*_ and $Z_{L}^{S}$ should usually have opposite signs (because the effects on longevity and disease are usually opposite), we use $\eta_{S}\;=\;{|Z}_{L}^{S}|$ because we don’t have directional information for some of the disease datasets. Dobriban et al. 2015[82] suggested that a natural choice for the standard error is $\sigma \;=\;\sqrt{N_{L}/N_{D}}$. The sample sizes for the diseases are often 10–20 times larger than the sample size for longevity, therefore we chose *σ* = .25 for simplicity. In principle, we could attenuate the value *η*_*S*_ towards zero because the effect on longevity is less direct than the effect on disease. However, there is no clear way to choose an attenuation factor. Instead, we use *σ* ^2^ which also weakens the connection between disease and longevity. We have made R code for calculating regularized weights available at https://github.com/edgardobriban/pvalue_weighting_r, and in the R package pweight, available from CRAN: https://cran.r-project.org/web/packages/pweight. After calculating the weight vector *w*, we applied it to the P values from the longevity GWAS *P*_*i*_, and controlled the false discovery rate with the Benjamini Hochberg step up procedure. From the set of all significant SNPs (FDR < 10%), we chose the lead SNP for each LD block (from DistiLD[61]) to be the SNP associated with the lowest FDR.

### Replication analysis of independent genetic loci

The lead SNP for each independent genetic locus that we discovered to be associated with human longevity was replicated in independent cohorts. Up to four independent cohorts were available to replicate each lead SNP (some SNPs were missing from some studies). Different types of P value were provided in each replication study (see Methods section on longevity cohorts), including: two-sided P values calculated from a fixed-effects meta-analysis, two-sided P values calculated under an allelic model, and two-sided P values calculated using the Wald test.

For each SNP in each study, we calculated one-sided replication P values to test whether the effect direction in the replication data *e*_*r*_ matched the effect direction in the discovery data *e*_*d*_:

$$p_{one-sided}=\{\begin{array}{ll} p_{two-sided}/2 & if\;e_{r}=\;e_{d}\\ \;1-\;p_{two-sided}/2 & \;if\;e_{r}\neq e_{d} \end{array}$$

We then used Stouffer’s test (as implemented in METAL[62]) to combine the one-sided replication p values for each SNP in *k* studies into a single one-sided “combined p”:

$$p_{combined}\;=\;1-\Phi (\frac{\sum_{i\;=\;1}^{k}Z_{i}}{\sqrt{k}})$$

where $Z_{i}\;=\;\Phi^{-1}(1-p_{i})$

Meta-analysis P values were corrected for multiple-testing with the Benjamini-Liu step-down procedure, which controls the false discovery rate and is recommended when testing a small number of independent hypotheses[63].

For replication, some SNPs were not genotyped in SICS and NECS, and so we used high quality proxy SNPs (R^2^ > 0.8, D’ = 1). To identify proxy SNPs, we used the Broad Institute SNAP tool[64], and selected the unique SNP with highest R^2^ in two reference populations: 1000 Genomes[58] and HapMap release 22[65] (S4 Table). In NECS, imputation results were also available for four SNPs in a set of 919 long-lived individuals (including the 801 in the NECS study), and a reduced set of 387 controls (a subset of the 914 in NECS)(S5 Table). Because proxy SNPs use more controls than imputed SNPs, we chose to use P values for proxy SNPs in our replication analyses (Table 3). This choice impacts the replication of rs514659; the proxy SNP rs505922 (R^2^ = 1, D’ = 1) is nominally significant in NECS, but the imputed P value is not (S5 Table).

In Table 2, the SNPs that show an association with longevity have small effects. With the exception of rs2075650, none of the individual SNPs explain more than 1% of the variance in the longevity phenotype. The rs2075650 SNP in APOE/TOMM40 explains about 1.2% of the variance in the longevity phenotype.

### SNP annotation data

*eQTL mapping*. We tested whether longevity-implicated SNPs influenced gene expression in the blood using an eQTL dataset incorporating peripheral blood samples from over 5000 individuals[37]. Here we report only those eQTL associations found to be significant at a false discovery rate < 5% in the original study.

*Other annotation data*. SNPs were mapped to gene names with SCAN[66] and to linkage disequilibrium blocks with DistiLD[61]. Proxies for lead SNPs were identified with the web tool SNAP[64], using both HapMap release 22[65] and 1000 Genomes Pilot 1[58] CEU genotype data. Locus plots were generated with LocusZoom[67].

### Ethics Statement

This study was approved by the Stanford University Institutional Review Board (approval number: 30051). No consent was received because this was a retrospective study on &gt;1 million people using summary, de-identified statistics from public databases.

## Supporting Information

S1 Text Deriving optimal P value weights. Methods used to derive the P value weighting scheme used in iGWAS.
(DOCX)

S1 Fig GWAS that show genetic overlap with exceptional longevity. As in Fig 1, the blue lines show the P values for longevity of the top 100, 250, and 500 SNPs from independent genetic loci, and the red lines show the background distribution of longevity P values. (*) P < 0.05, (**) P < 0.005.
(PDF)

S2 Fig GWAS that do not show genetic overlap with exceptional longevity. As in Fig 1, the blue lines show the P values for longevity of the top 100, 250, and 500 SNPs from independent genetic loci, and the red lines show the background distribution of longevity P values. (*) P < 0.05, (**) P < 0.005.
(PDF)

S3 Fig Regional plots for longevity-associated SNPs identified by iGWAS (NECS). Green boxes indicate genes harboring missense SNPs in LD with candidate longevity SNPs, and magenta boxes genes for which a candidate longevity SNP is an eQTL.
(PDF)

S4 Fig Regional plots for longevity-associated SNPs identified by iGWAS (90PLUS). Green boxes indicate genes harboring missense SNPs in LD with candidate longevity SNPs, and magenta boxes genes for which a candidate longevity SNP is an eQTL.
(PDF)

S1 Table Nine diseases show genetic overlap with longevity. For each of the 9 disease datasets showing a genetic overlap with longevity, we provide hypergeometric P values and fold enrichment, and identities of disease SNPs that are nominally associated with longevity (from the top 100).
(XLSX)

S2 Table 98 SNPs representing 8 unique genetic loci are significant in a longevity GWAS weighted by disease. (XLSX)

S3 Table eQTL results for all longevity SNPs, at a false discovery rate < 5%. (XLSX)

S4 Table Proxy SNPs used for replication, when data for lead SNP was missing. The best proxy SNP such that R2 > 0.8 and D’ = 1 was identified using data from two reference populations, 1000 Genomes and HapMap release 22 (Broad Institute SNAP tool[64]).
(XLSX)

S5 Table Imputation results for candidate longevity SNPs in NECS. (XLSX)
