# Supplementary material for: Genome-Wide Scan Informed by Age-Related Disease Identifies Loci for Exceptional Human Longevity
Source: PLoS Genet. 2015 Dec 17;11(12):e1005728. doi: 10.1371/journal.pgen.1005728 (PMC4683064; doi:10.1371/journal.pgen.1005728)
Supplement: S1 Text — Methods used to derive the P value weighting scheme used in iGWAS. (DOCX) [file pgen.1005728.s001.docx]

**Supplementary Note: Deriving optimal P-value weights**

**A1: Notation**

Our weights rely on the standard normal probability density function$\theta\left( x \right)={e^{{-x^{2}}/2}}/\sqrt{2\pi}$ and the normal cumulative distribution function $\Phi\left( x \right)=\int_{-\infty}^{x} \theta\left( y \right)dy$. We will denote a random variable $X$ that is normal with mean $\mu$ and variance $\sigma^{2}$ as $X\sim N\left( \mu,\sigma^{2} \right)$.

We will derive the weights for one-sided tests. By flipping signs if necessary we can assume that we are testing for negative prior effect sizes.

**A2: Statistical Model**

We assume that our association test statistics are standard normal with standardized effect sizes $\mu_{i}$ and variance 1: $T_{i}\sim N\left( \mu_{i},1 \right)$, where the index ranges from 1 to J . This can always be achieved by standardizing the test statistics. Further, we model the effect sizes as normal with prior effect size $\eta_{i}$ and variance $\sigma_{i}^{2}$: $\mu_{i}\sim N\left( \eta_{i},\sigma_{i}^{2} \right)$. Then it follows that the marginal distribution of $T_{i}$ is also normal, with parameters that are only functions of $\eta_{i}$ and $\sigma_{i}^{2}$: $T_{i}\sim N\left( \eta_{i},\sigma_{i}^{2}+1 \right)$. We also assume that the prior standard errors are strictly positive, i.e. $\sigma_{i}>0$, which corresponds to the realistic setting where there is some uncertainty in the prior information about $\mu_{i}$. The case $\sigma_{i}=0$ is treated in Roeder and Wasserman (2009)^1^, and the extension to positive variances is our main methodological contribution. We denote the marginal variance $\gamma_{i}^{2}:=\sigma_{i}^{2}+1$. The P-value *P_i_* for the one-sided test $\mu_{i}=0$ versus $\mu_{i}<0$ equals $P_{i}=\Phi\left( T_{i} \right)$.

**A3: Optimization Problem**

Many different approaches can be used to assign weights from a pleiotropic data set to be used in a second data set. For example, a candidate gene approach simply assigns equal weight to the candidate genes and zero to all other genes, and the conditional false discovery method assigns a weight according to the p-value in the pleiotropic data set. We want to find the weights that maximize the expected number of significant hits, given the prior information. We optimize the weights for the weighted Bonferroni procedure. This procedure can be defined for an arbitrary weight vector $w$, and for a fixed level $q$ it declares significant those SNPs whose p-values are small enough that $P_{i}\leq qw_{i}$. If the weights are non-negative and average to 1, then the weighted Bonferroni procedure controls the family-wise error rate at the level $a=Jq$.

Hence the number of significant hits as a function of the weights can be written formally as

$$R\left( w \right)=\sum_{i=1}^{J} I\left( \Phi\left( T_{i} \right)\leq qw_{i} \right)$$

Here $I\left( . \right)$ denotes the indicator function, which equals 1 if the statement between the parentheses is true and equals 0 otherwise. The expected number of significant hits, as a function of $w$, then equals by linearity of expectation:

$$E_{\mu}E_{T}R\left( w \right)=\sum_{i=1}^{J} P_{\mu,T}\left( \Phi\left( T_{i} \right)\leq qw_{i} \right)$$

The expectation is taken over two sources of uncertainty: The distribution of the test statistics $T_{i}$from the current data, and the distribution of the effect size $\mu_{i}$ estimated from the prior data. The above function can be written explicitly in terms of the normal cumulative distribution function. Thus, we have formulated the optimization objective explicitly. With this notation the optimization objective is to maximize the above function, subject to the constraints

$$w_{i}\geq0,\sum_{i=1}^{J} w_{i}=J$$

Next we discuss an efficient solution for the optimization problem.

**A4: Solution of the Optimization Problem**

Our solution of the optimization problem relies on Lagrange duality, which is a standard tool in numerical optimization. We refer the reader to any of a number of standard reference books on numerical optimization, eg.^2^. In short, in Lagrangian duality one reduces a constrained optimization problem to an unconstrained penalized optimization problem. Under certain conditions, if one has zero duality gap, then the penalized problems can be used to obtain the solution of the original problem. In our case the last step consists of a simple one-dimensional line search.

For convex problems, it is often easy to show that Lagrange duality works^2^. However, our problem is not convex, and for such problems zero duality gap holds only in a handful of cases, and relies on a detailed case-by-case analysis for each individual problem. We undertake such an analysis below, and for the full details we refer to the methodology paper Dobriban et al 2015^4^..

To summarize: Even though the optimization problem is not convex, we can solve it efficiently via a one-dimensional line search. The key reason is that under certain conditions the problem has zero duality gap.

To explain this formally, we need to introduce some additional notation. We define a function $c_{1}$, which will turn out to give the critical values for our test. $c_{1}=c_{1}\left( \eta,\gamma;\lambda\right)$ is a function of the prior mean and standard error $\eta,\gamma$ as well as of a so-called dual variable $\lambda$ which is of key importance in the optimization procedure. The function $c_{1}$ is given explicitly by the formula:

$$c_{1}\left( \eta,\gamma;\lambda\right)=\frac{-\eta+\sqrt{\eta^{2}+\left( \gamma^{2}-1 \right)\left( \eta^{2}+2\gamma^{2}log\left( \gamma\lambda\right) \right)}}{\gamma^{2}-1}$$

This formula arises from the analysis of the Lagrangian, the penalized version of the objective function mentioned above. The condition under which we can maximize the objective explicitly is given by an inequality involving the prior parameters $\eta_{i},\gamma_{i}$

$$\sum_{i=1}^{J} \Phi\left( c_{1}\left( \eta_{i},\gamma_{i};\lambda=1 \right) \right)\geq Jq$$

This is a mild condition, which holds in all cases involving GWAS data that we have seen. It can also be checked easily. After some analysis, one can see that it is enough that some prior effect sizes are large in magnitude; the details are presented in Dobriban et al 2015^4^.

Then we can state the following:

**Theorem.** If the above condition holds, then the optimal weights are, for a certain constant $\lambda$, $w_{i}=\Phi\left( c_{1}\left( \eta_{i},\gamma_{i};\lambda\right) \right)/q$. The constant $\lambda$ is the unique value for which $\sum_{i=1}^{J} w_{i}=J$, and it can be found via a one-dimensional line search.

We present a proof of this statement in Dobriban et al 2015^4^. It leads to an efficient algorithm for finding the optimal weights: 1) check the condition, 2) perform a one-dimensional line search starting at $\lambda=1$ for the unique value $\lambda$ such that the values $w_{i}=\Phi\left( c_{1}\left( \eta_{i},\gamma_{i};\lambda\right) \right)/q$ sum to $J$.

The one-dimensional line search is easy, because the weights depend monotonically on $\lambda$. Specifically, the function $f\left( \lambda\right)=\sum_{i=1}^{J} \Phi\left( c_{1}\left( \eta_{i},\gamma_{i};\lambda\right) \right)$ is monotonically decreasing in $\lambda$. Hence there are many standard numerical algorithms for the line search. The bisection method is the simplest one, and it has well-known convergence guarantees, se eg. Burden and Faires^3^. By numerical experiments we have also found that the classical Newton’s method is effective, and can be faster than the bisection method. However, it is not known theoretically that it converges, because it lacks the strong convexity required by most analyses. In our implementation, the user can choose either method.

We provide an open-source and free implementation of our method written in Matlab and R. Both implementations are available at <https://github.com/dobriban>. We also developed an R package, pweight, implementing p-value weighting methods and iGWAS. Pweight is available from CRAN:  <https://cran.r-project.org/web/packages/pweight/>.

References

1. Roeder, K. & Wasserman, L. Genome-Wide Significance Levels and Weighted Hypothesis Testing. *Stat. Sci.*  **24,** 398–413 (2009).

2. Boyd, S. & Vandenberghe, L. *Convex Optimization*. (Cambridge University Press, 2004).

3. Burden, R. L. & Faires, J. D. *Numerical analysis*. (Thomson : Brooks/Cole, 2005).

4. Dobriban, E., Fortney, K., Kim, S. & Owen, A. *Biometrika*. in press (2015).
