# Supplementary figures and images for: Genome-Wide Scan Informed by Age-Related Disease Identifies Loci for Exceptional Human Longevity
Source: PLoS Genet. 2015 Dec 17;11(12):e1005728. doi: 10.1371/journal.pgen.1005728 (PMC4683064; doi:10.1371/journal.pgen.1005728)

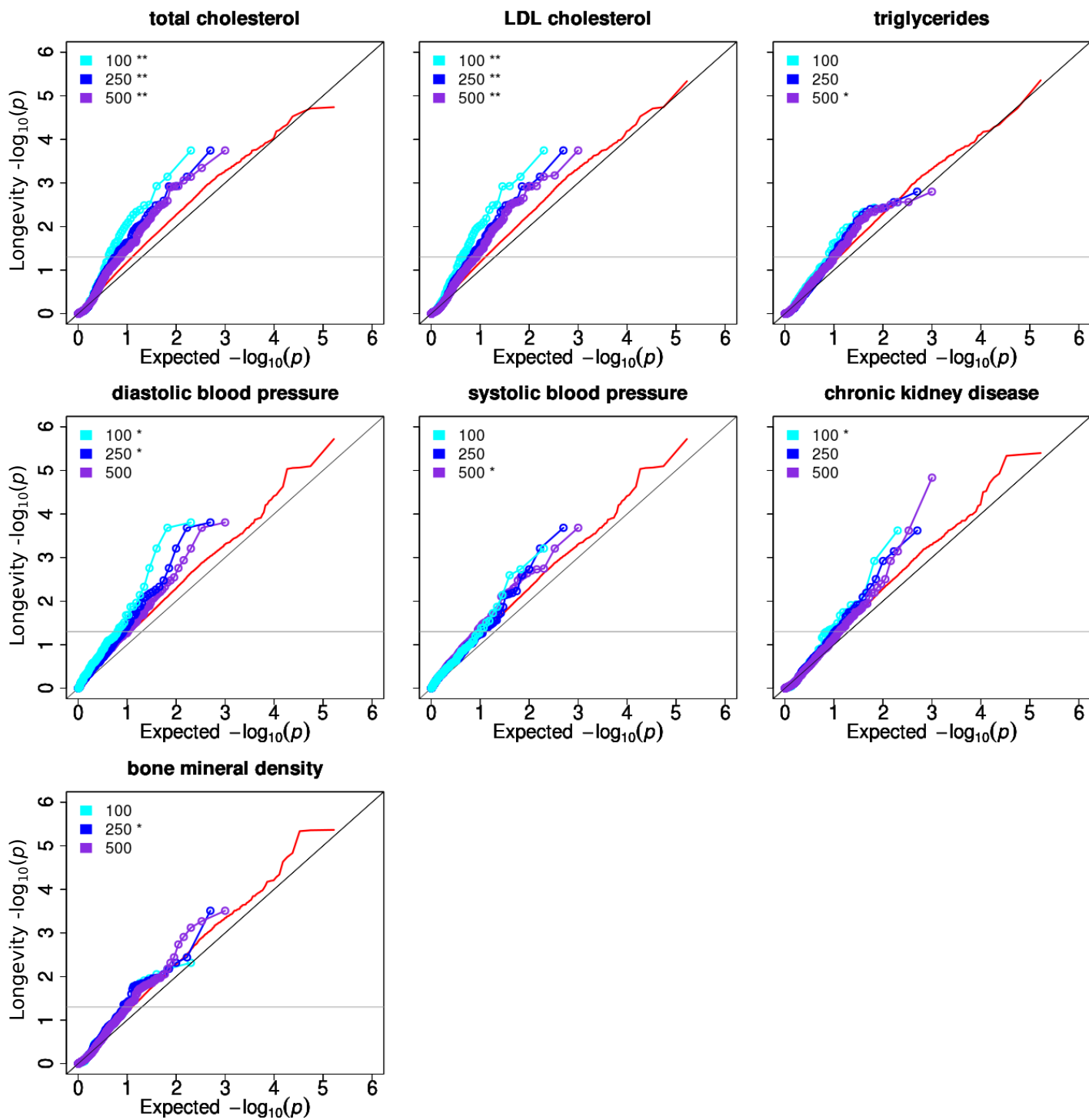

Supplement: S1 Fig — As in Fig 1, the blue lines show the P values for longevity of the top 100, 250, and 500 SNPs from independent genetic loci, and the red lines show the background distribution of longevity P values. (*) P < 0.05, (**) P < 0.005. (PDF) [file pgen.1005728.s002.pdf]

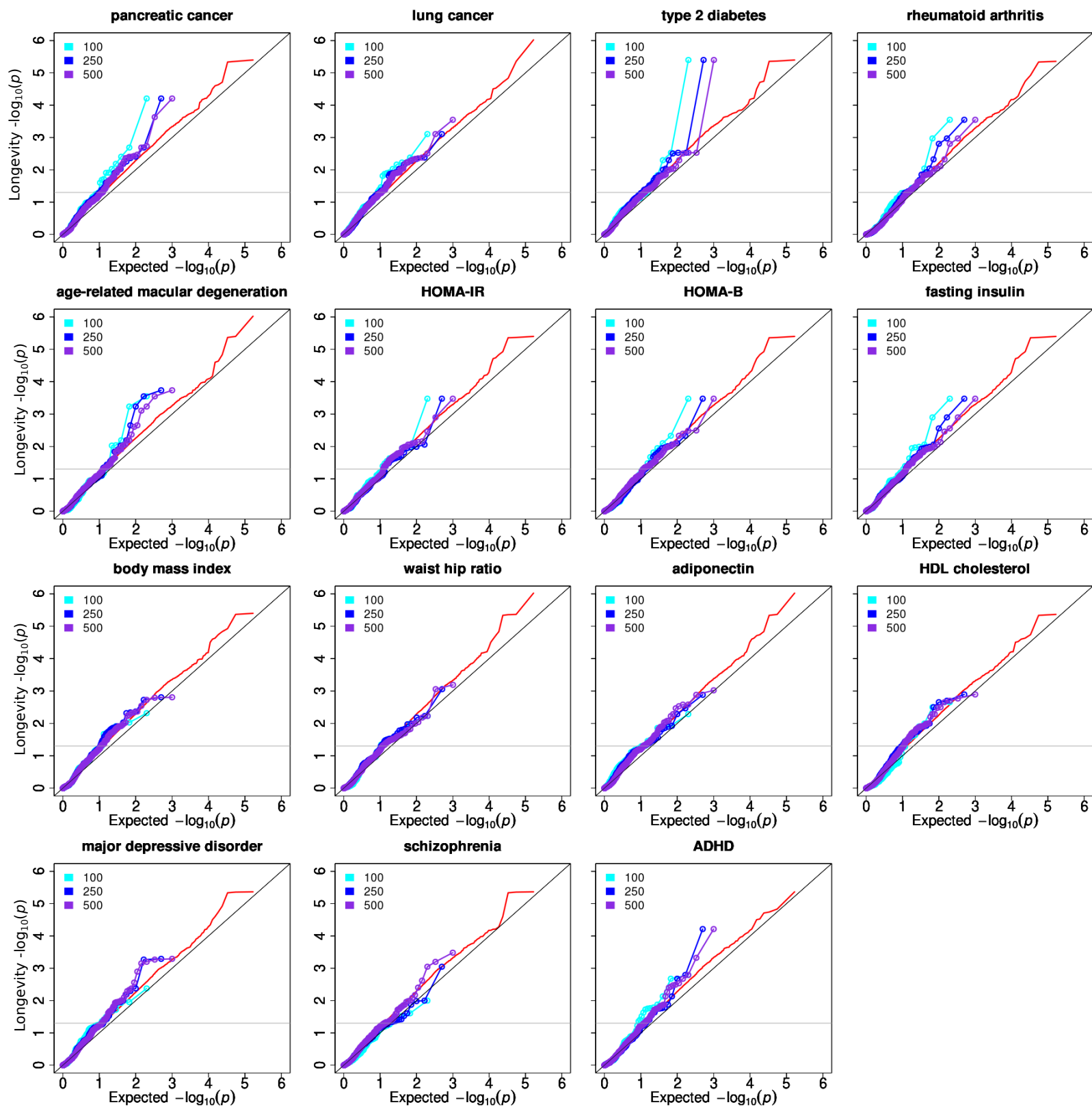

Supplement: S2 Fig — As in Fig 1, the blue lines show the P values for longevity of the top 100, 250, and 500 SNPs from independent genetic loci, and the red lines show the background distribution of longevity P values. (*) P < 0.05, (**) P < 0.005. (PDF) [file pgen.1005728.s003.pdf]

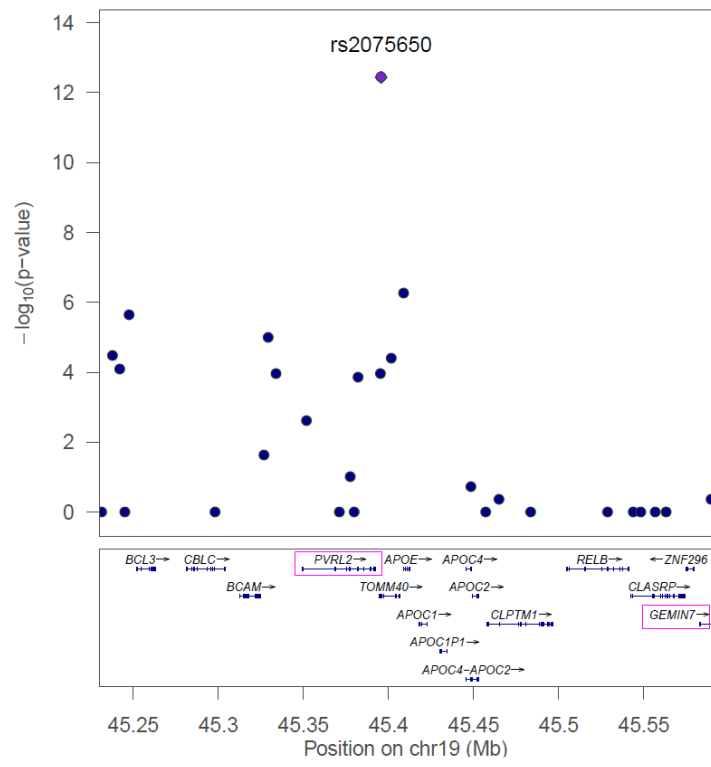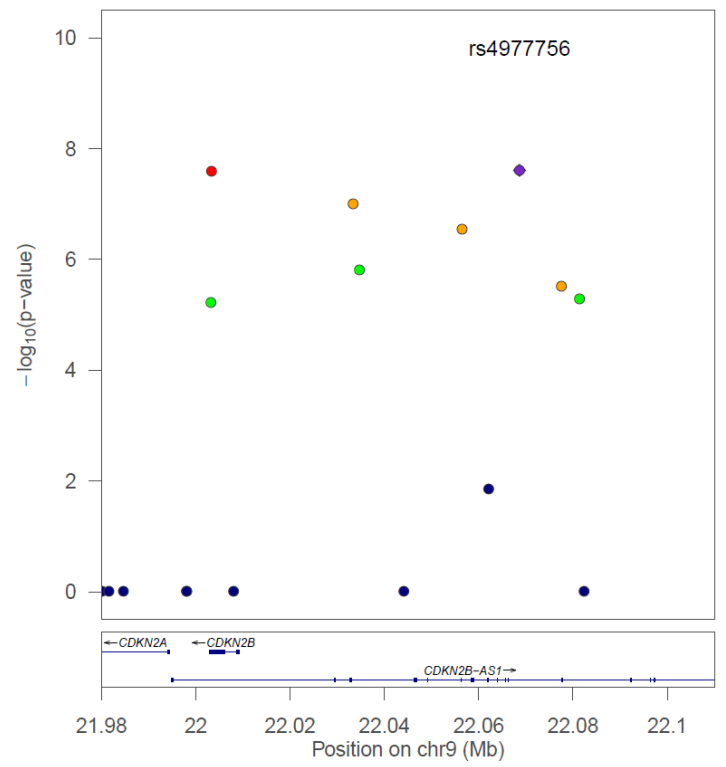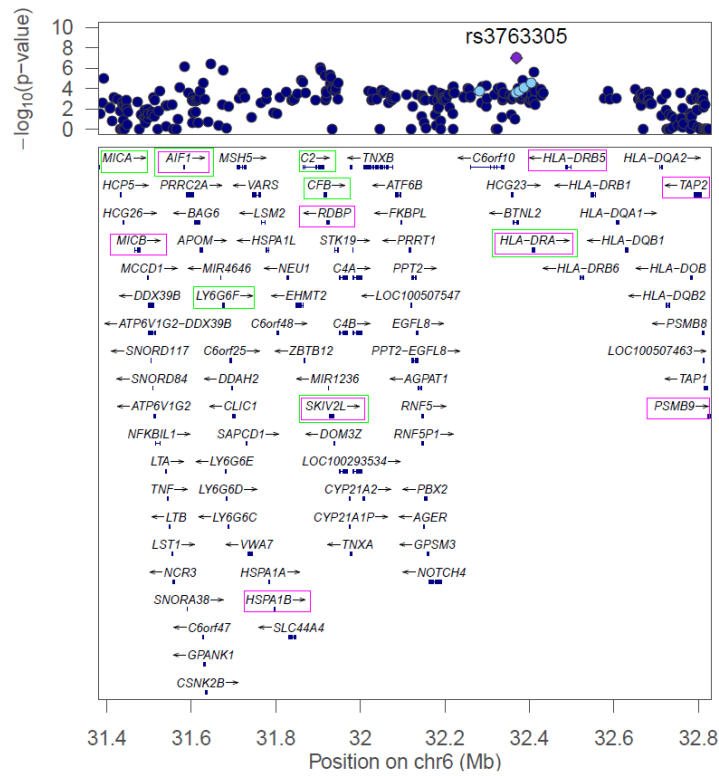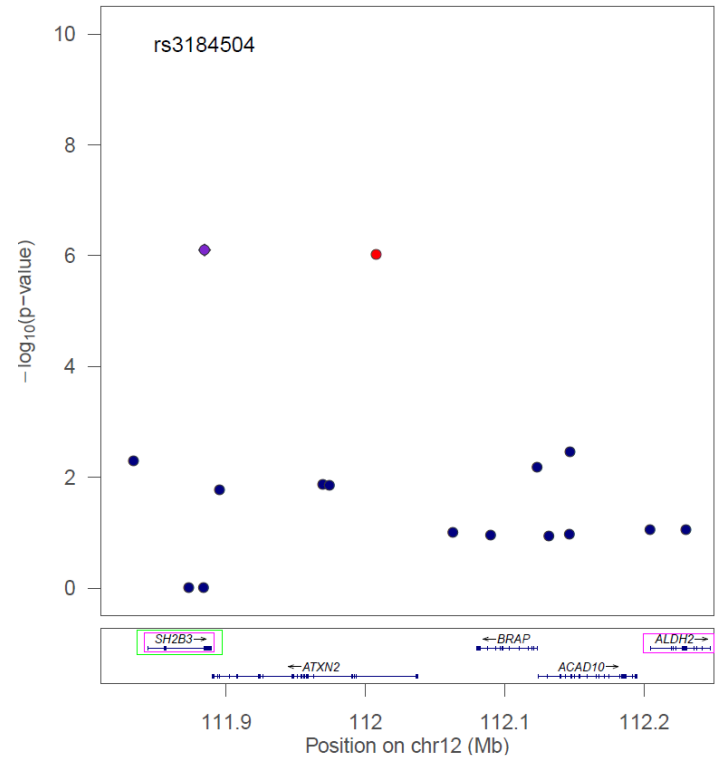

Supplement: S3 Fig — Green boxes indicate genes harboring missense SNPs in LD with candidate longevity SNPs, and magenta boxes genes for which a candidate longevity SNP is an eQTL. (PDF) [file pgen.1005728.s004.pdf]

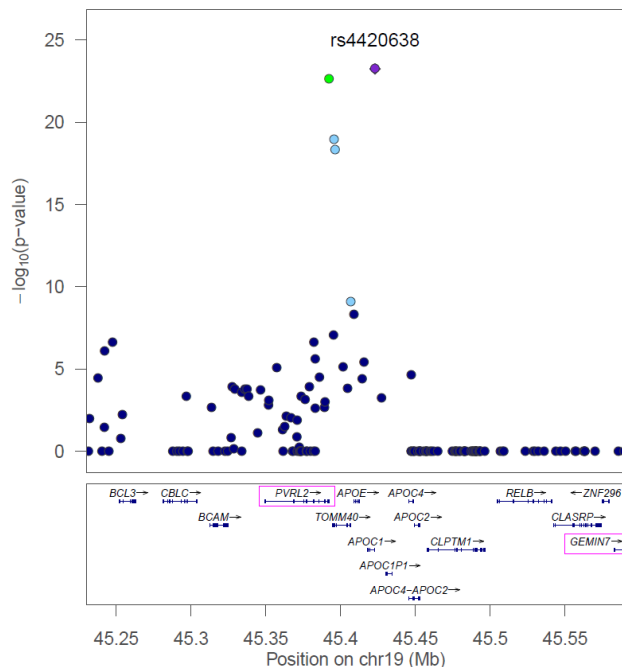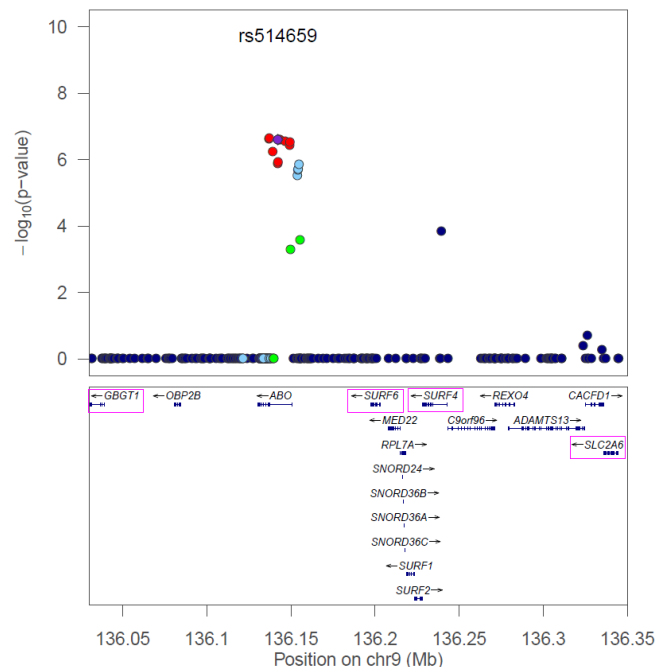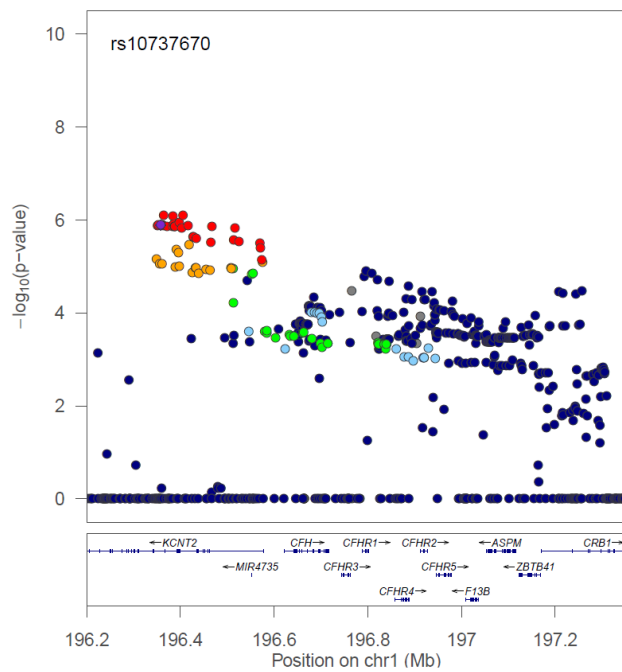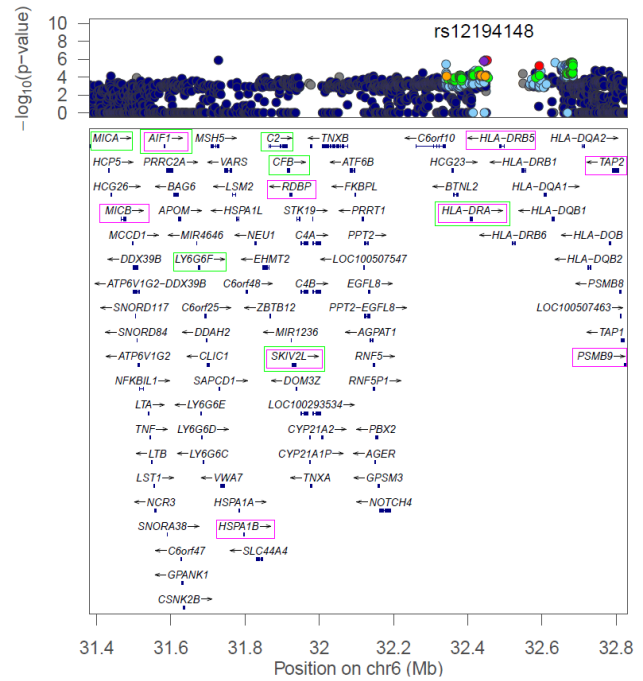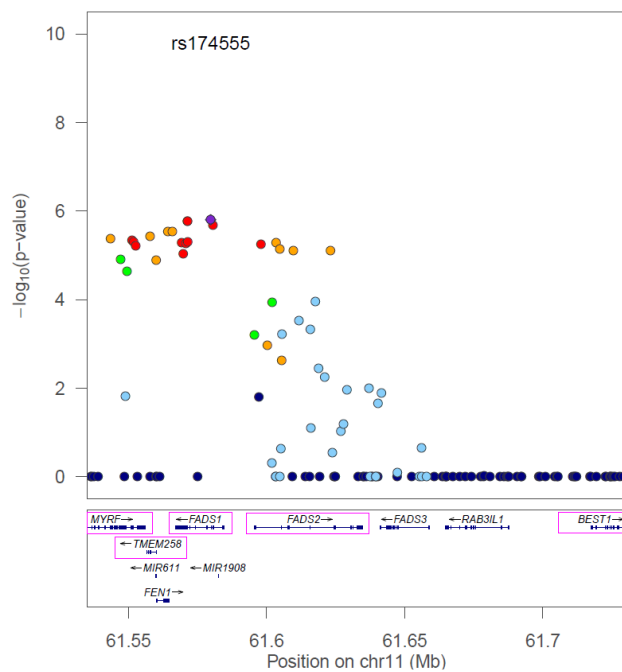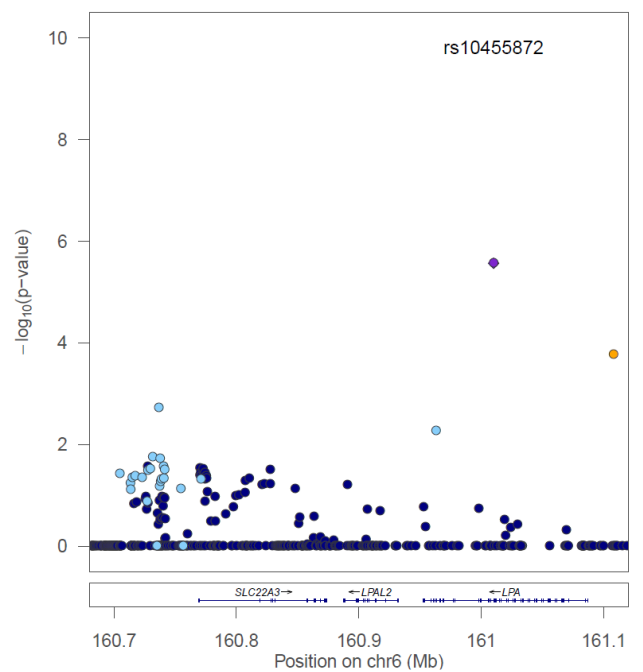

Supplement: S4 Fig — Green boxes indicate genes harboring missense SNPs in LD with candidate longevity SNPs, and magenta boxes genes for which a candidate longevity SNP is an eQTL. (PDF) [file pgen.1005728.s005.pdf]
